# Phylogenomic assessment of 23 equid alphaherpesvirus 1 isolates obtained from USA-based equids

**DOI:** 10.1186/s12985-023-02248-z

**Published:** 2023-11-29

**Authors:** Ugochi Emelogu, Andrew C. Lewin, Udeni B. R. Balasuriya, Chin-Chi Liu, Rebecca P. Wilkes, Jianqiang Zhang, Erinn P. Mills, Renee T. Carter

**Affiliations:** 1 Veterinary Clinical Sciences, School of Veterinary Medicine, Department of Veterinary Clinical Sciences, Louisiana State University, Baton Rouge, LA 70803 USA; 2 School of Veterinary Medicine, Louisiana Animal Disease Diagnostic Laboratory, Baton Rouge, LA USA; 3 Purdue University, Animal Disease Diagnostic Laboratory, West Lafayette, IN USA; 4 Iowa State University, Veterinary Diagnostic Laboratory, Ames, IA USA

**Keywords:** Equine alphaherpesvirus 1, Equine, Herpes Virus, Genome, Recombination, Phylogeny, Nucleotide substitution

## Abstract

**Background:**

Equid alphaherpesvirus 1 (EHV-1) is a global viral pathogen of domestic equids which causes reproductive, respiratory and neurological disease. Few isolates acquired from naturally infected USA-based hosts have been fully sequenced and analyzed to date. An ORF 30 (DNA polymerase) variant (A2254G) has previously been associated with neurological disease in host animals. The purpose of this study was to perform phylogenomic analysis of EHV-1 isolates acquired from USA-based hosts and compare these isolates to previously sequenced global isolates.

**Methods:**

EHV-1 was isolated from 23 naturally infected USA-based equids (6 different states, 15 disease outbreaks) with reproductive (22/23) or neurological disease (1/23). Following virus isolation, EHV-1 DNA was extracted for sequencing using Illumina MiSeq. Following reference-based assembly, whole viral genomes were annotated and assessed. Previously sequenced EHV-1 isolates (n = 114) obtained from global host equids were included in phylogenomic analyses.

**Results:**

The overall average genomic distance was 0.0828% (SE 0.004%) for the 23 newly sequenced USA isolates and 0.0705% (SE 0.003%) when all 137 isolates were included. Clade structure was predominantly based on geographic origin. Numerous nucleotide substitutions (mean [range], 179 [114–297] synonymous and 81 [38–120] non-synonymous substitutions per isolate) were identified throughout the genome of the newly sequenced USA isolates. The previously described ORF 30 A2254G substitution (associated with neurological disease) was found in only one isolate obtained from a host with non-neurological clinical signs (reproductive disease), six additional, unique, non-synonymous ORF 30 substitutions were detected in 22/23 USA isolates. Evidence of recombination was present in most (22/23) of the newly sequenced USA isolates.

**Conclusions:**

Overall, the genomes of the 23 newly sequenced EHV-1 isolates obtained from USA-based hosts were broadly similar to global isolates. The previously described ORF 30 A2254G neurological substitution was infrequently detected in the newly sequenced USA isolates, most of which were obtained from host animals with reproductive disease. Recombination was likely to be partially responsible for genomic diversity in the newly sequenced USA isolates.

**Supplementary Information:**

The online version contains supplementary material available at 10.1186/s12985-023-02248-z.

## Background

Equid alphaherpesvirus 1 (EHV-1), a varicellovirus of the *Herpesviridae* family, is a widespread pathogen of equids which is capable of causing respiratory, ocular, reproductive (i.e., abortion), neurological disease (equine herpes myeloencephalopathy, EHM) and neonatal death [1–5]. First isolated from abortion material in 1933 [6], EHV-1 is now recognized as a major threat to the global equine industry [7]. In particular, neurological disease in host equids represents a serious welfare and economic problem and has therefore been the focus of numerous prior investigations [8–11].

EHV-1 is a double stranded DNA virus with an approximately 150 kilobase genome consisting of 76 unique open reading frames (ORF) and 4 duplicated repeat regions [12]. Previous studies have indicated that ORF 30 (DNA polymerase) substitution A2254G (leading to amino acid variation N752D) is associated with neurological disease in host animals [9, 13–15]. Although the underlying mechanism for this is unclear, it has been suggested that EHV-1 with the A2254G substitution replicates at a higher level and induces a longer-lasting viraemia than isolates without this substitution [16]. Notably, isolates recovered from host animals with neurological disease do not consistently possess this substitution [17]. Although prior work has found that certain isolates are more capable of inducing abortion in experimental studies [18, 19], EHV-1 genetic determinants for abortion have not been identified. The ORF 68 region (which encodes a non-essential membrane associated component) has previously been utilized as a genetic marker for grouping EHV-1 isolates [20].

Although several phylogenomic assessments of naturally occurring EHV-1 have been previously performed, limited numbers of viral isolates obtained from USA-based host animals have been included in these analyses [17, 21, 22]. Ongoing phylogenomic investigation of existing isolates is likely to improve global surveillance and subsequent control of this virus. The purpose of this study was to perform phylogenomic analysis of EHV-1 isolates acquired from USA-based hosts, and to compare these isolates to previously sequenced global isolates. Based on previous work, we hypothesized that isolates would form clades based on geographic origin [17] and that the previously described ORF 30 A2254G substitution would be present only in isolates obtained from host animals with neurological disease [20].

## Methods

### Viral isolates and hosts

Twenty-three archived EHV-1 isolates obtained over a 24-year period (1997–2021) from naturally infected, USA-based domestic equids were sequenced in this study. Isolates from host animals located in 6 different US states (i.e., California, Iowa, Indiana, Virginia, North Dakota and South Dakota) were included. The viral isolates sequenced in this study (with associated host information, where available) are shown in Table 1.

**Table 1:** Viral isolate and associated host data for the 23 EHV-1 isolates sequenced (whole viral genome) for the present study

Isolate	Genbank Accession ID	Host State	Collection Date	Isolation Date	Tissue source	Host Age	Host Species	Host Sex	Disease Type	ORF30 A > G, 2254	Synonymous Substitutions	Non-Synonymous Substitutions
LS0433182	OR085515	South Dakota	Unknown	9/1/2004	Unknown	8 years	Equus caballus	Female	Neurological	No	138	62
LS050627	OR085496	North Dakota	Unknown	6/27/2005	Unknown	Unknown	Unknown	Unknown	Reproductive	Yes	135	71
LS080124	OR085497	Indiana	Unknown	1/24/2008	Unknown	Unknown	Unknown	Unknown	Reproductive	No	174	86
LS100106	OR085498	Indiana	Unknown	1/6/2010	Unknown	Unknown	Unknown	Unknown	Reproductive	No	174	86
LS110097	OR085499	Iowa	3/25/2011	11/7/2021	Liver	Fetal	Unknown	Unknown	Reproductive	No	174	87
LS110114	OR085500	Iowa	4/6/2011	11/7/2021	Lung	Fetal	Unknown	Unknown	Reproductive	No	115	58
LS110976	OR085501	Iowa	3/25/2011	11/7/2021	Spleen	Fetal	Unknown	Unknown	Reproductive	No	174	87
LS111457	OR085502	Iowa	4/6/2011	11/7/2021	Liver	Fetal	Unknown	Unknown	Reproductive	No	132	43
LS113812	OR085503	Iowa	2/4/2011	11/7/2021	Liver	Fetal	Unknown	Unknown	Reproductive	No	157	83
LS119764	OR085504	Iowa	3/25/2011	11/7/2021	Lung	Fetal	Unknown	Unknown	Reproductive	No	116	49
LS130922	OR085505	Iowa	1/8/2013	3/4/2013	Unknown	Fetal	Unknown	Unknown	Reproductive	No	174	71
LS140310	OR085506	Indiana	Unknown	3/10/2014	Unknown	Unknown	Unknown	Unknown	Reproductive	No	236	111
LS140325	OR085507	Indiana	Unknown	3/25/2014	Unknown	Unknown	Unknown	Unknown	Reproductive	No	158	85
LS143101	OR085508	Indiana	Unknown	3/10/2014	Unknown	Unknown	Unknown	Unknown	Reproductive	No	228	115
LS161221	OR085509	Indiana	Unknown	12/21/2016	Unknown	Unknown	Unknown	Unknown	Reproductive	No	191	88
LS161227	OR085510	Indiana	Unknown	12/27/2016	Unknown	Unknown	Unknown	Unknown	Reproductive	No	227	91
LS170109	OR085511	Indiana	Unknown	1/9/2017	Unknown	Unknown	Unknown	Unknown	Reproductive	No	261	104
LS170307	OR085512	Indiana	Unknown	3/7/2017	Unknown	Unknown	Unknown	Unknown	Reproductive	No	145	93
LS180416	OR085513	Indiana	Unknown	4/16/2018	Unknown	Unknown	Unknown	Unknown	Reproductive	No	297	120
LS213627	OR085514	Iowa	4/22/2021	6/8/2021	Lung	Unknown	Unknown	Unknown	Reproductive	No	126	52
LS9719959	OR085516	Virginia	Unknown	4/15/1997	Unknown	Fetal	Equus caballus	Unknown	Reproductive	No	114	38
LS9816616	OR085517	California	Unknown	3/16/1998	Unknown	Fetal	Equus caballus	Male	Reproductive	No	294	112
LS9922376	OR085518	Virginia	Unknown	5/5/1999	Unknown	Fetal	Equus caballus	Female	Reproductive	No	184	77

Note that complete host data was not available in most cases. ORF30 A > G, 2254 refers to a previously described substitution which has been associated with neurological disease [20]

A total of 114 additional EHV-1 isolate genomes which had previously been fully/near fully sequenced were obtained from Genbank (https://www.ncbi.nlm.nih.gov/genbank/) for inclusion in phylogenomic analyses. These isolates were obtained from hosts located in four global regions including Europe (84/114), USA (6/114), Australia (11/114) and Asia (13/114). A list of these isolates can be found in Additional file 1: Table S1.

### Cell culture and viral isolation

CCL-57 cells (Equine dermal cells, ATCC CCL-57, Manassas, VA) were cultured at 37 °C/5% CO_2_ in T25 flasks (Thermo Fisher, Waltham, MA, USA) using Dulbecco’s modified Eagle medium (DMEM) (Thermo Fisher, Waltham, MA, USA) fortified with 10% fetal bovine serum (FBS) (Thermo Fisher, Waltham, MA, USA) and 1% penicillin/streptomycin (Thermo Fisher, Waltham, MA, USA) until 90–100% confluent.

Approximately 100µL of each viral stock (virus previously isolated using various cell lines for archival) was added to 1 mL of DMEM fortified with 2% FBS and 1% penicillin/streptomycin sulfate and briefly vortexed before being added to individual T25 flasks containing confluent CCL-57 cells. Flasks were then placed on a rocker at room temperature for 60 min, after which an additional 4 mL of DMEM fortified with 2% FBS and 1% penicillin/streptomycin sulfate was added to each flask before incubation at 37 °C and 5% CO_2_. Flasks were then monitored daily for visible cytopathic effect (CPE). Once 100% CPE was verified, three cycles of freezing at − 80 °C and thawing at room temperature were carried out. The contents of each flask were transferred to a 15 ml conical tube and centrifuged at 400 × g for 5 min at 4 °C. The resultant supernatant was then transferred to cryotubes and immediately stored at − 80 °C pending DNA extraction.

### Viral DNA extraction, viral species confirmation and DNA concentration assessment

Using 200µL of stored cell culture supernatant, viral DNA extraction was carried out using a commercially available kit (Purelink Viral DNA Mini Kit, Thermo Fisher, Waltham, MA, USA). DNA concentration was determined using a Qubit dsDNA HS Assay Kit (Life Technologies, Grand Island, NY). Prior to full viral genome sequencing, real-time polymerase chain reaction (7900HT Fast Real-Time PCR System, Applied Biosystems) was carried out on samples to confirm viral identity, using EHV-1 specific primers and probe [23].

### Illumina genomic DNA sequencing

Exact DNA concentrations for all samples were used to prepare sequencing libraries using the Nextera XT DNA Library Prep Kit (Illumina Inc. San Diego, CA, USA) and Nextera XT Index Kit v2 (Illumina Inc. San Diego, CA, USA). Final library quality and quantity were determined using a Fragment Analyzer Instrument (Fragment Analyzer System, Agilent). Libraries were pooled following indexing, prior to sequencing. Paired-end whole genome sequencing was performed using an Illumina MiSeq instrument using a 600 cycles MiSeq Reagent Kit v3 (Illumina Inc. San Diego, CA, USA).

### Genome assembly and alignments

Reference-based genome (V592, Genbank accession number of AY464052) assembly was carried out using Geneious Prime (version 2020.2.4), as previously described [24, 25]. In brief, paired end reads were first trimmed using BBDuk Adapter/Quality trimmer version 38.84 (right end, Kmer length = 27, maximum substitution = 1, minimum quality = 30, minimum overlap = 20, minimum length = 30). The trimmed paired end reads were then mapped to the reference genome using Geneious Prime. A consensus sequence was generated from the aligned reads with gaps filled with “N’s”. Each genome was annotated using annotation similarity transfer within Geneious Prime, prior to submission to the online Genbank data repository.

Viral genomes were aligned as previously described [24, 25], using MAFFT alignment tool (MAFFT ver 7.490), with default parameters [26]. Multiple alignments (with or without an equid alphaherpesvirus 8 (EHV-8) outgroup, Genbank accession NC017826) were created to include all of the newly sequenced USA isolates with or without previously sequenced isolates (obtained from Genbank, shown in Additional file 1: Table S1). Sites with at least 20% gaps were stripped from the alignments using the ‘Mask Alignment’ tool in Geneious Prime for subsequent phylogenomic analyses.

### Nuclotide subsitution analysis

Nucleotide substitution analysis was performed as previously described [24, 25] using the ‘Geneious Variant Finder’ (Geneious Prime version 2020.2.4, minimum coverage = 100, minimum variant frequency = 0.25, maximum variant *p* value = 10^–6^). Substitutions were identified by comparing the EHV-1 sequenced isolates to the reference genome (V592).

### Phylogenomic and recombination analysis

Phylogenomic analysis of whole viral genomes was performed as previously described [24, 25]. ModelFinder [27], within IQ-Tree 2 version 1.6.12 [28], was used for automatic selection of the best-fit model (K3Pu + F + R9) for the stripped alignment containing all available EHV-1 isolates and an EHV-8 outgroup (NC017826). The resultant treefile was viewed using Splitstree (version 4.16.1) [29] and Geneious Prime. Pairwise genomic distances were determined using EHV-1 alignments (without an EHV-8 outgroup) in MEGA11 (ver. 11.0.13) [30] with the gamma distribution model, partial deletion of gaps and 1000 bootstrap replicates.

Recombination analysis was performed using RDP version 4.100 [31] on an alignment containing all 137 EHV-1 genomes (without an EHV-8 outgroup) using manual bootscan (window = 1200, step = 500, replicates = 100, 70% cutoff, Jin and Nei model [32]), RDP [33], GENECONV [34], MaxChI [35], Chimaera [36] and Siscan [37].

## Results

### Viral Isolates, host information and sequencing results

The whole genome of 23 EHV-1 isolates from 23 host animals in six US states were sequenced; 10/23 from Indiana, 8/23 from Iowa, 2/23 from Virginia, 1 each from California, North Dakota, and South Dakota (Table 1). Isolates were collected and archived over a period of 24 years between 1997 and 2021. In total, isolates from 15 separate outbreaks (same geographic location, isolated or collected within 30 days of other isolate(s)) were included. Host data was included, where available. The age of 11/23 hosts and species (*Equus caballus*) of 4/23 hosts was known, while sex of 3/23 hosts was known (Table 1). Host disease data was available for all 23 isolates (Table 1). Of the 23 host animals, 22/23 had reproductive disease (including abortion and stillbirth) while 1/23 had neurological disease (ataxia and urinary incontinence).

Additional file 2: Table S2 shows sequencing details for the 23 newly sequenced EHV-1 isolates. The total number of reads obtained ranged from 340,648 (LS9922376) to 1,946,622 (LS140310). The average number of mapped reads ranged from 69,798 for strain LS110976 to 452,971 for strain LS143101. Mean genome coverage was 240X, with a range of 62.1X (LS110097) to 607.9X (LS143101). The overall mean genome GC content was 55.3%, with a range of 54.3% (LS050627) to 66.5% (LS110097). The average mapped genome length was 149,920 with a range of 148,164 (LS110097) to 151,048 (LS180416).

### Phylogenomic analysis

**Fig. 1:**
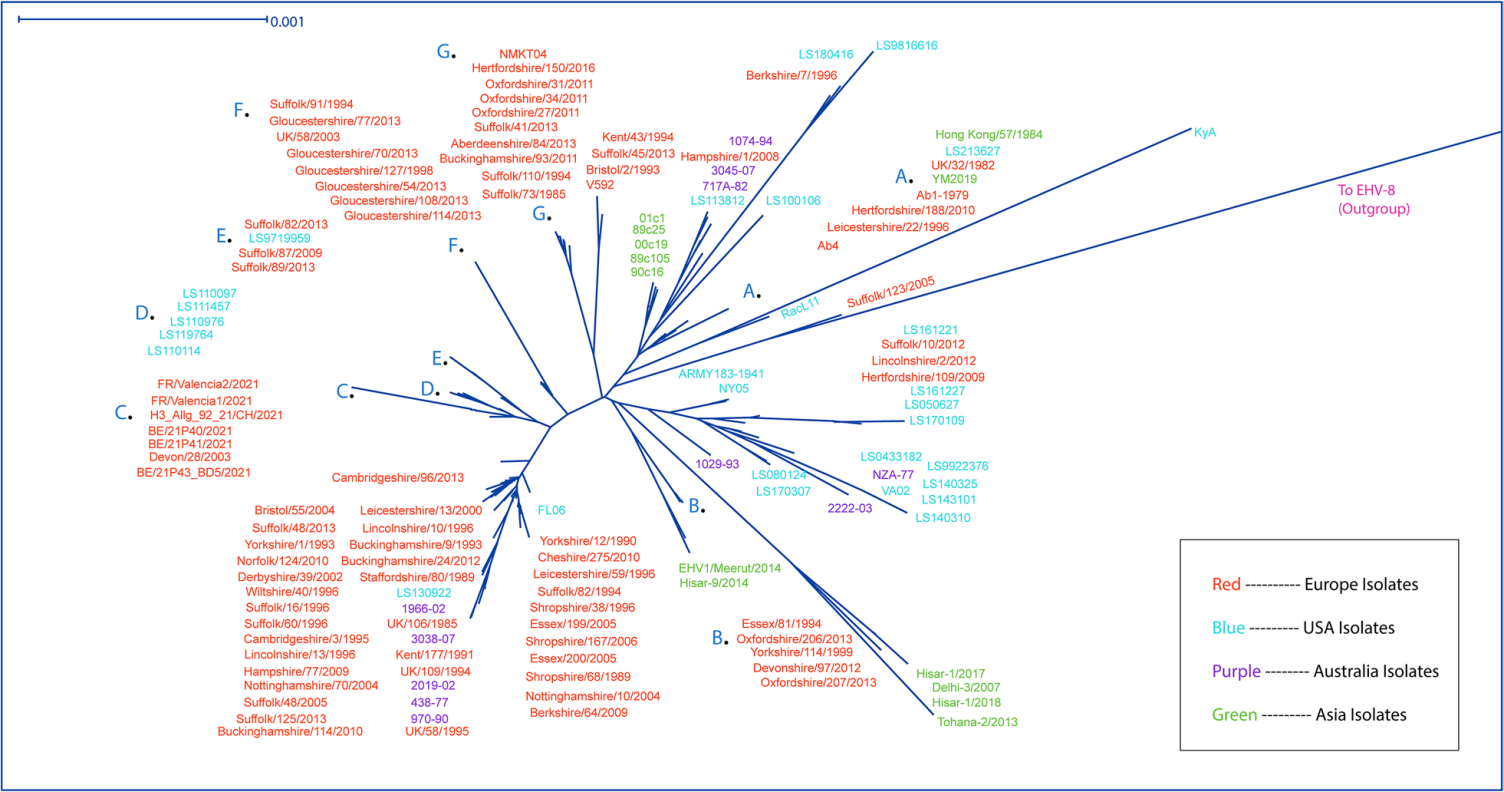
Phylogenomic tree (viewed with Splitstree (ver. 4.16.1) [29]) showing 137 EHV-1 isolates including the 23 EHV-1 isolates sequenced for the present study, with an EHV-8 outgroup (shown in pink, Genbank accession NC017826). Diagram created using an alignment subsequently modeled using ModelFinder [27] in Iqtree (ver. 1.6.12) [28]. The letters** A** to **G** are used to indicate the position of large groups of isolates at a single point on the diagram. European, USA, Australian and Asian isolates are shown in red, blue, purple and green respectively. Evidence of isolate grouping by regional origin (Europe, USA, Australia or Asia) is evident, as is grouping of isolates which were obtained from individual outbreaks (e.g. clade **D**)

**Fig. 2:**
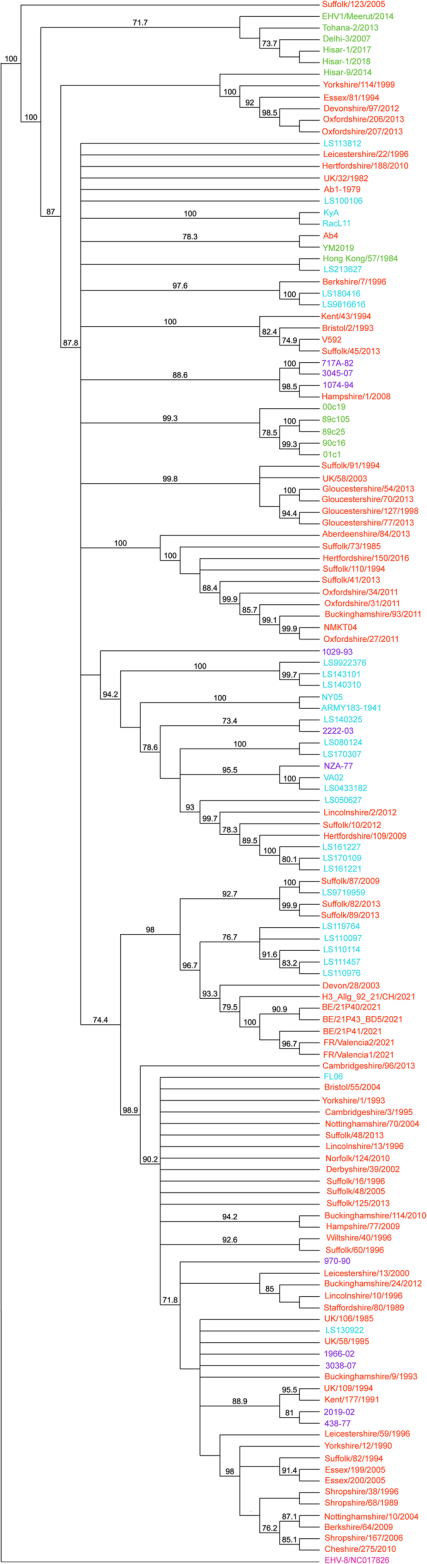
Maximum likelihood tree (viewed in Geneious Prime (ver. 2020.2.4)) showing 137 EHV-1 isolates including the 23 EHV-1 isolates sequenced for the present study, with an EHV-8 outgroup (shown in pink, Genbank accession NC017826). Diagram created using an alignment subsequently modeled using ModelFinder [27] in Iqtree (ver. 1.6.12) [28]. Branch labels represent bootstrap values which exceeded 70%. Evidence of isolate grouping by regional origin (Europe, USA, Australia or Asia) is evident, as is grouping of isolates which were obtained from individual outbreaks

The phylogenomic relationships between all 137 (114 previously sequenced, 23 newly sequenced) sequenced EHV-1 isolates (with an EHV-8 outgroup) are shown in Fig. 1. A maximum likelihood tree, generated using the same treefile, is shown in Fig. 2. Newly sequenced USA based isolates were widely distributed among existing clades, with some grouping observed between certain isolates obtained from the same disease outbreak. For example, 10 newly sequenced isolates from Indiana were analyzed, obtained from 6 separate disease outbreaks. Of these 6 outbreaks, 2 outbreaks yielded 3 isolates each; one in 2014 (LS140310, LS140325 and LS143101) and the other in 2016/2017 (LS161221, LS161227 and LS170109). Isolates from each outbreak were found to be mostly identical, as expected, and clustered closely together. Of the 6 isolates from a single outbreak in Iowa in 2011, 5/6 of these were mostly homologous and clustered together (LS110097, LS110114, LS110976, LS111457 and LS119764), with the remaining isolate (LS113812) found at a distant position (see ‘Distance Analysis’, below). Although many of the 137 isolates grouped according to geographic origin (Europe, USA, Australia or Asia), numerous exceptions were observed (Figs. 1 and 2).

### Distance analysis

The overall average genomic distance was 0.0828% (SE 0.004%) for the 23 newly sequenced USA isolates and 0.0705% (SE 0.003%) when all 137 isolates were included. Interregional genomic distances are shown in Table 2. Overall, the interregional distances as shown in Table 2 were broadly similar. The lowest distance difference was between isolates from Australia and Europe (0.0592%) and the greatest distance difference was between Asia and United States (0.0913%). Intraregional genomic distances were; 0.0828% (SE 0.004%) for USA isolates, 0.0784% (SE 0.004%) for Asian isolates, 0.0580% (SE 0.003%) for European isolates and 0.0574% (SE 0.004%) for Australian isolates.

**Table 2:** Mean interregional genomic distances of isolates obtained in Europe, USA, Asia or Australia

	Europe	USA	Asia	Australia
Europe		0.00392	0.00407	0.00341
USA	0.0813		0.00425	0.00399
Asia	0.0816	0.0913		0.00426
Australia	0.0592	0.0750	0.0803	

The lower left values are the interregional distance values (expressed as percentages) and the upper right values are the corresponding standard error values

Of the six Iowa isolates (LS110097, LS111457, LS113812, LS110976, LS119764 and LS110114) that originated from the same outbreak, five isolates clustered together (LS110097, LS111457, LS110976, LS119764 and LS110114) with a relatively low intraclade distance of 0.00763% (SE 0.00173%). When the remaining isolate from the same outbreak was considered (LS113812), inter-isolate distance was approximately 5 × greater; 0.0367% (SE 0.00389%).

### Nucleotide substitution detection

Nucleotide substitutions (relative to the reference genome, V592) were detected in all 23 newly sequenced EHV-1 isolates. On average, 179 (range 114–297) synonymous and 81 (range 38–120) non-synonymous substitutions were identified in each genome (Table 1). Nucleotide substitutions were relatively evenly distributed throughout the EHV-1 genomes. Details of substitutions in each of the 23 newly sequenced EHV-1 isolates can be found in Additional file 3: Tables S3 to S25.

Following whole genome substitution detection in each of the 23 newly sequenced EHV-1 isolates, genomes were specifically assessed for the previously described ORF 30 A2254G substitution, which has been associated with host neurological disease [20] (Table 1). This substitution was present in only 1/23 newly sequenced isolates (LS050627), which was obtained from a host equid with reproductive disease located in North Dakota in 2005. The genome of the single newly-sequenced isolate (LS0433182) from a host animal with confirmed neurological disease did not contain the ORF 30 A2254G substitution. In addition to the ORF 30 A2254G substitution, a total of 6 additional, unique non-synonymous ORF 30 substitutions were identified in the 23 newly sequenced USA isolates (Table 3). Most (22/23) isolates contained at least 1 (range 0–3) of these 6 unique non-synonymous substitutions. These 6 unique non-synonymous ORF 30 substitutions included; A2279G (D760G), A2968G (K990E), A1984C (S662R), G1286A (R429K), T2149C (F717L) and C3131T (S1044L). Five unique synonymous ORF 30 substitutions were identified in the 23 newly sequences USA isolates which included; T924C, G96A, G2805T, G2874A and C2352T.

**Table 3:** Synonymous and non-synonymous ORF30 (DNA polymerase) substitutions in each of the 23 newly sequenced USA EHV-1 isolates

Isolate	ORF 30 synonymous substitutions		ORF 30 non-synonymous substitutions		
	ORF location	Polymorphism	ORF location	Polymorphism	AA change, position
LS0433182	924	T > C	2279	A > G	D to G, 760
			2968	A > G	K to E, 990
LS050627	924	T > C	2254	A > G	N to D, 752
			2968	A > G	K to E, 990
LS080124	924	T > C	1984	A > C	S to R, 662
			2968	A > G	K to E, 990
LS100106	924	T > C	2968	A > G	K to E, 990
LS110097	96	G > A	2968	A > G	K to E, 990
	924	T > C			
	2805	G > T			
LS110114	96	G > A	2968	A > G	K to E, 990
	924	T > C			
	2805	G > T			
LS110976	96	G > A	2968	A > G	K to E, 990
	924	T > C			
	2805	G > T			
LS111457	96	G > A	2968	A > G	K to E, 990
	924	T > C			
	2805	G > T			
LS113812	924	T > C	2968	A > G	K to E, 990
LS119764	96	G > A	2968	A > G	K to E, 990
	924	T > C			
	2805	G > T			
LS130922	96	G > A	2968	A > G	K to E, 990
	924	T > C			
	2874	G > A			
LS140310	924	T > C	1286	G > A	R to K, 429
			2968	A > G	K to E, 990
LS140325	924	T > C	2968	A > G	K to E, 990
LS143101	924	T > C	1286	G > A	R to K, 429
			2968	A > G	K to E, 990
LS161221	924	T > C	2968	A > G	K to E, 990
LS161227	924	T > C	2968	A > G	K to E, 990
LS170109	924	T > C	2968	A > G	K to E, 990
LS170307	924	T > C	1984	A > C	S to R, 662
			2968	A > G	K to E, 990
LS180416	924	T > C	2968	A > G	K to E, 990
	2352	C > T			
LS213627	924	T > C	2968	A > G	K to E, 990
LS9719959	96	G > A	N/A		
	924	T > C			
LS9816616	924	T > C	2149	T > C	F to L, 717
			2968	A > G	K to E, 990
			3131	C > T	S to L, 1044
LS9922376	924	T > C	2968	A > G	K to E, 990

Polymorphisms: A = adenine, G = guanine, C = cytosine, T = thymine. Amino acid abbreviations: D = aspartic acid, G = glycine, K = lysine, E = glutamic acid, N = asparagine, S = serine, R = arginine, F = phenylalanine, L = leucine

### Recombination analysis

Genomic evidence of recombination was present in most (22/23) of the 23 newly sequenced EHV-1 isolates, when assessed using RDP4 [33]. Manual bootscan assessment detected evidence of recombination involving at least 1 other EHV-1 isolate in 20/23 newly sequenced isolates (Additional file 4: Table S26). The mean number of possible recombinants per isolate was 16 (range 0–53) based on manual bootscan assessment. When the complete RDP analysis was utilized, most (16/23) isolates were found to demonstrate evidence of recombination by at least one of the 5 additional methods of detection (RDP, GENECONV, MaxChi, Chimaera, Siscan); details can be found in Additional file 5: Table S27. Only 1 isolate (LS050627) was found to demonstrate no evidence of recombination by any of the methods utilized. As noted above (see Nucleotide Substitution Detection), this isolate was obtained in 2005 from an equid host located in North Dakota with reproductive disease and possessed the previously described ORF 30 A2254G neurological substitution.

## Discussion

Phylogenomic and recombinational assessment of the 23 newly sequenced EHV-1 isolates obtained from USA-based hosts revealed many similarities with previously sequenced global isolates. Phylogenomic assessment of previously sequenced EHV-1 isolates and related alphaherpesviruses have been reported, facilitating comparison with the results presented herein.

As previously described [17], the genome of EHV-1 isolates obtained from multiple hosts involved in the same disease outbreak were found to share a high degree of similarity. This finding is not unexpected and has previously been reported in studies of a similar nature for related alphaherpesviruses such as canid alphaherpesvirus 1 (CHV-1) [24] and felid alphaherpesvirus 1 (FHV-1) [25]. Within our sample set, multiple isolates from three notable outbreaks of reproductive disease were included: Iowa in 2011, Indiana in 2014 and 2016. In all 3 cases, most isolates from the same outbreak (with the exception of LS113812, Iowa 2011) were found to be near-identical. While isolates obtained from hosts in the same geographic region (USA, Asia, Australia or Europe) clustered together, there were numerous exceptions to this pattern which was observed. For example, newly sequenced USA isolates clustered with varying combinations of isolates from distant geographic locations, including Europe, Australia and Asia. While small variations in pairwise interregional genomic distances were detected, it is clear that EHV-1 isolate geographic origin cannot be determined solely on sequence data. In contrast, FHV-1 isolates obtained from widespread geographic locations mostly do form clades based on geographic origin [38]. Although the definitive reason for this discrepancy is unknown, possible reasons include sample size, degree of viral intraspecies conservation and host species differences in global animal (or animal product) movements. Although previous assessments [20, 39, 40] have sought to utilize EHV-1 ORF 68 as the primary method to classify isolates into clade structures, we chose to perform viral full genome sequencing to identify nucleotide substitutions throughout the genome, as has been previously performed [17] and described.

Since being described almost 20 years ago [20], the ORF 30 A2254G substitution has been investigated in the context of equine herpes myeloencephalopathy (EHM) [2, 9, 13–15, 17, 41, 42]. Although the substitution has been shown to be significantly associated with neurological disease [20], notably the substitution is not present in all isolates recovered from hosts with EHM [17]. As the underlying mechanism for this association between this specific substitution and EHM is unknown, the reasons for this apparent inconsistency are presently unknown. Most (22/23) of the newly sequenced USA isolates included in the present study were obtained from host animals with reproductive disease, with only 1 originating from a host equid with neurological disease. As noted above, only 1/23 isolates possessed the ORF 30 A2254G substitution, which originated from a host with reproductive disease. The single isolate obtained from the host animal with neurological disease did not possess the ORF 30 A2254G substitution. Inclusion of a higher number of isolates originating from hosts with neurological disease in future studies of similar design are suggested.

Several other ORF 30 substitutions have been described, in addition to the well-known ORF 30 A2254G substitution. Certain EHV-1 isolates (obtained from animals with reproductive disease) have been noted to contain a non-synonymous substitution in ORF 30 at position 2258 (A2258C) [43]; this substitution was not detected in any of the isolates in our sample. In addition, a recently described non-synonymous ORF 30 substitution at position 2254 (A2254C) has been described and documented in isolates from the USA and France [44–46]; again, this substitution was not detected in any of the isolates in our sample. In addition to the ORF 30 A2254G substitution, we detected 6 other unique non-synonymous substitutions in the 23 newly sequenced isolates, some of which have been previously described [17, 20]. Surveillance using assays targeting this highly conserved region is likely to be beneficial for use in future outbreaks. The effect of each of these substitutions on virulence, if any, is unknown.

Both intraspecies and interspecies recombination have been shown to be prevalent mechanisms of diversity in EHV-1 and related alphaherpesviruses [17, 24, 25, 38, 47–51]. We detected evidence of recombination in most (22/23) of the newly sequenced EHV-1 isolates in the present study. It is unknown why one of the newly sequenced isolates (LS050627) did not demonstrate evidence of recombination, although this could represent the limitations of the predictive computational processes utilized for this purpose. Increased understanding of the mechanisms by which EHV-1 substitutions develop is likely to have significant implications for both disease surveillance and control in host equids.

The present study has several limitations, including sample size, incomplete host animal information, lack of host disease type diversity, and viral genome gaps following sequencing. The present study included 23 newly sequenced EHV-1 isolates from USA based host animals. While concerted attempts were made to include a higher number of isolates, sample availability was a limiting factor. Approximately 46 samples (suspected to contain either EHV-1 DNA or viable EHV-1) were initially screened, but only 23 yielded high quality EHV-1 DNA in quantities suitable for Illumina MiSeq sequencing. During sample collection, efforts were made to collect as much host animal data as possible. As many of the samples had been collected and archived many years prior, host information was not consistently readily available. Most (22/23) of the EHV-1 isolates were obtained from hosts with reproductive disease. Inclusion of a higher number of isolates from animals with neurological and/or respiratory disease may have facilitated assessment of relationships between viral genome substitutions and host disease type. Finally, in common with all Illumina platform viral genome sequencing assessments, the reference-based assembly of high GC content regions resulted in sequence gaps with the genome sequences of the isolates from this study. While unavoidable with this approach, this was accounted for during analysis and is therefore not expected to have affected the results or subsequent conclusions.

## Conclusions

Overall, the genomes of the 23 newly sequenced EHV-1 isolates obtained from USA-based hosts were similar to previously sequenced global isolates. The previously described ORF30 A2254G substitution was infrequently detected in the newly sequenced USA isolates, most of which were obtained from host animals with reproductive disease. In line with previous findings, recombination was likely to be partially responsible for genomic diversity in the newly sequenced USA isolates.

### Supplementary Information

**Additional file 1: Table S1**. Details of all 137 EHV-1 isolates used for this study including the 114 archived isolates obtained from Genbank.

**Additional file 2: Table S2**. Sequencing details of the 23 EHV-1 isolates obtained from USA based hosts, showing the number of reads, number of mapped reads, mean coverage, % GC content and mapped genome length.

**Additional file 3: Tables S3–S25**. Nucleotide substitution tables for the 23 newly sequenced 23 USA EHV-1 isolates. Details include substitution position and size, type and amino acid change (if any).

**Additional file 4: Table S26**. Detailed results of manual bootscan recombination detection performed using an alignment containing all 137 EHV-1 isolates in RDP version 4.100 (33) (window = 1200, step = 500, replicates = 100, Jin and Nei model). Bootstrap support values of 70% or greater were considered reliable evidence of possible recombination. Evidence of recombination events was detected in most (20/23) of the newly sequenced EHV-1 isolates by manual bootscan.

**Additional file 5: Table S27**. Detailed results of recombination detection using an alignment containing all 137 EHV-1 isolates in RDP version 4.100 (33). Detection methods used (with default parameters) included R (RDP), G (GENECONV) (34), M (MaxChi) (35), C (Chimaera) (36) and S (Siscan) (37).

## Data Availability

The datasets generated and/or analyzed during the current study are available in the Genbank repository (https://www.ncbi.nlm.nih.gov/genbank/).

## Abbreviations

A: Adenine
ATCC: American Type Culture Collection
C: Cytosine
CCL: Cellosaurus cell line
CHV-1: Canine herpesvirus type 1
CPE: Cytopathic effect
D: Aspartic acid
DMEM: Dulbecco’s modified eagle medium
DNA: Deoxyribonucleic acid
E: Glutamic acid
EHM: Equine herpesvirus encephalomyelitis
EHV-1: Equine herpesvirus type 1
EHV-8: Equine herpesvirus type 8
F: Phenylalanine
FBS: Fetal bovine serum
FHV-1: Feline herpesvirus type 1
G: Guanine or glycine
K: Lysine
L: Leucine
MAFFT: Multiple alignment using fast fourier transform
MEGA: Molecular evolutionary genetics analysis
N: Asparagine
ORF: Open reading frame
R: Arginine
RDP: Recombination Detection Program
S: Serine
SE: Standard error
T: Thymine
US/USA: United States of America
